# The Yemeni Brown Algae *Dictyota dichotoma* Exhibit High *In Vitro* Anticancer Activity Independent of Its Antioxidant Capability

**DOI:** 10.1155/2020/2425693

**Published:** 2020-02-17

**Authors:** Amina El-Shaibany, Molham AL-Habori, Tareq Al-Maqtari, Hassan Al-Mahbashi

**Affiliations:** ^1^Department of Pharmacology, Faculty of Pharmacy, University of Sana'a, Sana'a, Yemen; ^2^Department of Biochemistry & Molecular Biology, Faculty of Medicine and Health Sciences, University of Sana'a, Sana'a, Yemen; ^3^Department of Forensic Medicine & Clinical Toxicology, Faculty of Medicine and Health Sciences, University of Sana'a, Sana'a, Yemen

## Abstract

The aim of this study was to investigate the anticancer and antioxidant activities as well as the safety of the brown algae *Dictyota dichotoma* of the Western seacoast of Yemen. Cytotoxicity of methanol extract of *D. dichotoma* and several of its fractions, petroleum ether, chloroform, ethyl acetate, n-butanol, and aqueous extracts against seven different cancer cell lines was determined by crystal violet staining. The antioxidant activity was also assessed using the DPPH radical scavenging assay. Acute toxicity study was performed on rats at increasing doses of the methanol extract. Extracts of *D. dichotoma* exerted a significant dose-dependent cytotoxicity on the seven tumor cell lines but were generally more selective on MCF-7 and PC-3. Among all fractions, the chloroform fraction of the *D. dichotoma* displayed the highest cytotoxic activity and was most effective in MCF-7, PC3, and CACO cells (IC_50_ = 1.93 ± 0.25, 2.2 ± 0.18, and 2.71 ± 0.53 *μ*g/mL, respectively). The petroleum ether fraction was also effective, particularly against MCF-7 and PC-3 (IC_50_ = 4.77 ± 0.51 and 3.93 ± 0.51 *μ*g/mL, respectively) whereas the activity of the ethyl acetate fraction was more pronounced against HepG2 and CACO (IC_50_ = 5.06 ± 0.21 and 5.06 ± 0.23 *μ*g/mL, respectively). Of all the extracts tested, the crude methanolic extract of the algae exhibited only a modest antioxidant potential (IC_50_ = 204.6 ± 8.3 *μ*g/mL). Doses as high as 5000 mg/kg body weight of *D. dichotoma* methanolic extracts were safe and well tolerated by rats. The overall results showed that *D. dichotoma* exhibited a significant cytotoxic activity probably due to the occurrence of nonpolar cytotoxic compounds, which is independent of its antioxidant capability.

## 1. Introduction

Cancer is one of the leading causes of death worldwide; currently, the primary target of many research groups is to find novel anticancer drugs that boost chemotherapy treatment and decrease death rates [1]. The growing body of evidences substantiating the protective role of marine products in controlling chronic diseases such as cancer has motivated substantial scientific concern in recognizing the active secondary metabolites of marine products [2]. During the last few decades, a number of anticancer compounds have been identified from marine organisms [3]; however, only few chemotherapy drugs have been approved for clinical treatment [4], such as the antitumor drug trabectedin (ecteinascidin 743 isolated from the tunicate *Ecteinascidia turbinata*), eribulin (halichondrin B isolated from the sponge *Halichondria okadai*), and the macrolides lactones bryostatins (isolated from the brown bryozoan *Bugula neritina*) [1, 5, 6]. Trabectedin is used today for ovarian cancer and soft tissue sarcomas [7], while eribulin is used in the treatment of late-stage breast cancer [8].

Marine algae constitute a significant underexploited part of the diet and traditional remedies in the Eastern hemisphere. Because of their distinctive living environment, algae are rich in bioactive components such as phycocyanin, steroids, terpenoids, and polysaccharides [9, 10]. Marine macroalgae have been the center of much attention as a promising source of new affordable, safe, and effective anticancer agents [4]. Marine, green, brown, and red algae displayed a broad range of biological activities that are beneficial against cancer including cytotoxic, antioxidant, anti-inflammatory, and antimitotic activities [11, 12]. The anticancer activity of compounds derived from macroalgae demonstrated reduced cell viability and induction of apoptosis in cancer cells [10, 13, 14]. *Dictyota dichotoma* (*D. dichotoma*) and four other brown algae species: *Bifurcaria bifurcata*, *Cystoseira tamariscifolia, Desmarestia ligulata*, and *Halidrys siliquosa* have exhibited a substantial cytotoxic activity to three cancer cell lines: Daudi (human Burkitt's lymphoma), Jurkat (human leukemic T cell lymphoblast), and K562 (human chronic myelogenous leukemia) [15]. Moreover, the crude extract of *D. dichotoma* isolated from the Egyptian Red Sea coast exerted a potent cytotoxic effect on breast carcinoma tumor cell line MCF7 [16]. The complexity, poor prognosis, and patient specificity, type specificity, and stage specificity of cancer require the investigation and identification of novel compounds with effective clinical utility. With a long seacoast along the Red and the Arabian Seas, Yemen is acknowledged for its richness in numerous macroalgae species, with no available data regarding natural products from marine organisms and their application as traditional medications. The aim of this study was, therefore, to assess the anticancer potential (using crystal violet staining assay) on seven different tumor cell lines: colon cancer (HCT-116), breast cancer (MCF-7), hepatocellular carcinoma (HepG2), lung adenocarcinoma (A-549), prostate cancer (PC-3), cervical cancer (HeLa), and intestinal cancer (CACO); the antioxidant activity (using the DPPH radical scavenging activity assay) and the safety profile of *D. dichotoma* settled along these shorelines.

## 2. Materials and Methods

### 2.1. Algae Collection

*D. dichotoma* was collected from the seacoasts of Hodeida city, West of Yemen, in March 2015, and was authenticated by Dr. Abdulsalam Al-Kawri, Faculty of Marine Science and Environment, Hodeidah University, Yemen. A specimen (voucher no. 242) was deposited at the Pharmacognosy Department, Faculty of Pharmacy, Sana'a University.

### 2.2. Extraction and Fractionation of *D. dichotoma*

The dried and coarsely powdered algal material was extracted by maceration with 90% methanol for 3 weeks at room temperature. The combined obtained methanolic extract was filtered and concentrated under reduced pressure using a rotary evaporator to give a dark greenish-brown crude residue (MEA). The semisolid residue of the total extract was suspended in water and successively partitioned with petroleum ether, chloroform, ethyl acetate, n-butanol, and aqueous mother liquor. Each fraction was dried over anhydrous sodium sulphate and evaporated to dryness to yield: PEA (petroleum ether), CEA (chloroform), EEA (ethyl acetate), BEA (n-butanol), and AEA (remaining aqueous mother liquor) fractions.

### 2.3. Experimental Animals

Swiss Wister albino mice with an average weight of 25 g were used for the acute toxicity study. All animals were fed with standard animal feed and water *ad libitum*. The animals were acclimatized to the laboratory conditions for five days prior to experimentation. All experiments carried out were approved by the Institutional Ethical Committee, Faculty of Medicine and Health Sciences, Sana'a University (360-12/03/2015), and were conducted according to the standard guideline for the use of laboratory animals [17].

### 2.4. Acute Toxicity Study

The acute oral toxicity was conducted according to the Organization for Economic Co-operation and Development (OECD) guidelines [18]. Thirty-six rats were used and randomly assigned to 6 groups (6 animals per group). Animals were deprived of food but given water 16 hours prior to dosing. Methanolic extracts in Tween 80 (1% w/v) at a serial concentration of 100, 1000, 2500, 4000, and 5000 mg/kg body weight were then given orally to test groups, while the control group received the vehicle only at the same volume. Body weight, general physical conditions (appearance, fur and skin conditions, mucus membranes, and eyes), behavioral pattern, autonomic and neurological effects (salivation, diarrhea, tremors, convulsion, and lethargy), and mortality were observed after administration at the third hour on the first day and throughout the following 48 hours and then daily thereafter for 14 days [19]. At the end of the experiment, the animals underwent euthanasia with a high dose of thiopental (100 mg/kg IP) [20].

### 2.5. Crystal Violet Cytotoxicity Study

For the determination of cytotoxicity of the algal extracts [21], multiple cancer cell lines were selected to represent different types of cancers: HCT-116, MCF-7, HepG2, A-549, PC-3, HeLa, and CACO. Cells were obtained from the American Type Culture Collection (ATCC, Rockville, MD). Each tested cell line was seeded in 96-well flat-bottomed microtiter plates at a density of 1 × 10^4^ cells per well in 100 *μ*l of growth medium. Twenty-four hours later, fresh medium containing serial twofold dilutions of the algal extract (0.87, 1.56, 3.13, 6.25, 12.5, 25, 50, and 100 *μ*g/mL) was added to the confluent cell monolayers using a multichannel pipette (three wells per dilution). Petroleum ether, chloroform, ethyl acetate, and n-butanol extracts of *D. dichotoma* were added to the wells in triplicate. Dimethyl sulfoxide (DMSO) (<1%) was used as a solvent for all the tested extracts which showed no effect on the test. Doxorubicin HCl (Sigma-Aldrich) was used as reference drug positive control tested at the same concentrations (0.87, 1.56, 3.13, 6.25, 12.5, 25, 50, and 100 *μ*g/mL) used for the tested extracts. The plate was incubated at 37°C in a humidified incubator with 5% CO_2_. After 48 hrs, the number of viable cells was determined using the crystal violet colorimetric assay [22]. Briefly, the media were aspirated from wells and 1% of the crystal violet solution was added. Thirty minutes later, the solution was aspirated and the plates were rinsed using tap water until all excess stain is removed. Glacial acetic acid (30%) was then added to all wells with gentle shaking before measuring the absorbance of the wells at 590 nm via a microplate reader. The cell inhibitory concentration 50 (IC_50_) was estimated from graphic plots of dose-response data. All procedures and readings were performed in triplicate.

### 2.6. Antioxidant Assay

The antioxidant activity was determined using the 2,2-diphenyl-1-picrylhydrazyl (DPPH) free radical scavenging assay as described by [23]. The principle of the DPPH method lies in measuring the reduction of the DPPH radical in alcoholic solution by an H^+^-donating species. The absorbance of the DPPH radical without antioxidant (control) and the positive control (ascorbic acid) was also measured. All the determinations were performed in triplicate and then averaged.

### 2.7. Statistical Analysis

Results were expressed as mean ± Standard deviation (SD) of three triplicates. The data were analyzed using *Statistically Package for Social Sciences* (SPSS) version 21. Independent *T*-test was used to test the significance of the differences between groups. Differences between means were considered significant at *P* value of ≤0.05.

## 3. Results and Discussion

### 3.1. Cytotoxic Activity Against Cancer Cell Lines

**Figure 1:**
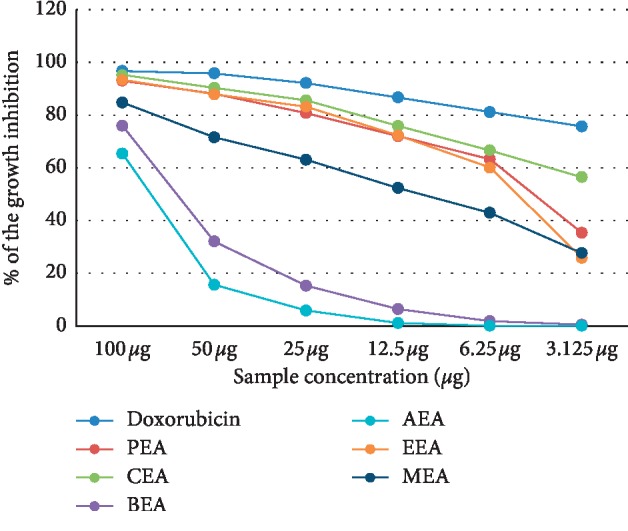
Percentage inhibition of cell growth of *D. dichotoma* extracts against the MCF-7 cell line. PEA (petroleum ether), CEA (chloroform), EEA (ethyl acetate), BEA (n-butanol), AEA (remaining aqueous mother liquor) fractions, and MEA (methanol).

**Figure 2:**
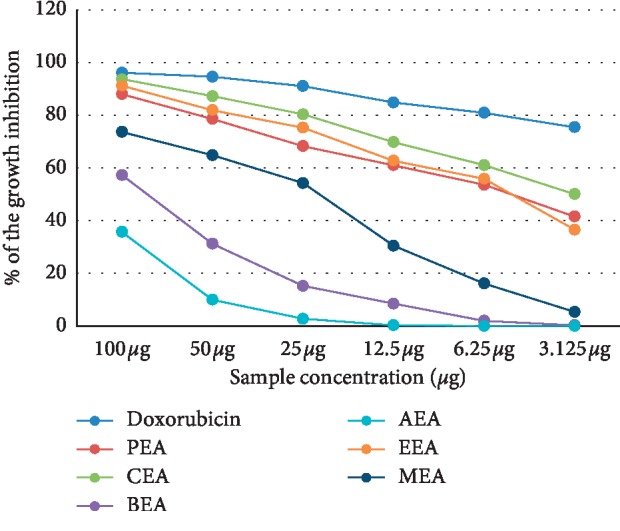
Percentage inhibition of cell growth of *D. dichotoma* extracts against the HCT-116 cell line. PEA (petroleum ether), CEA (chloroform), EEA (ethyl acetate), BEA ((n)-butanol), AEA (remaining aqueous mother liquor) fractions, and MEA (methanol).

**Figure 3:**
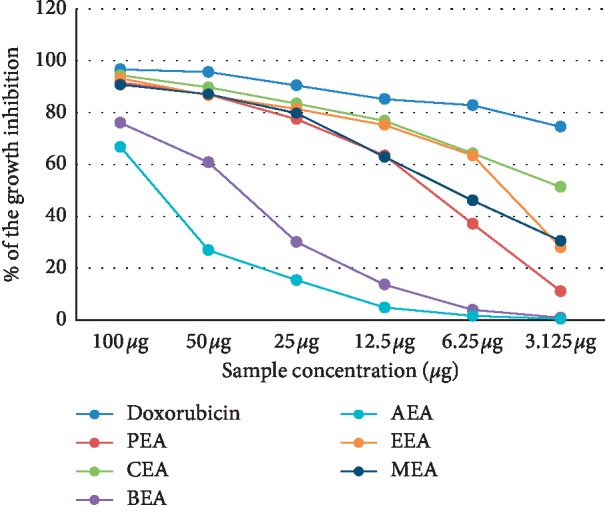
Percentage inhibition of cell growth of *D. dichotoma* extracts against the HepG-2 cell line. PEA (petroleum ether), CEA (chloroform), EEA (ethyl acetate), BEA ((n)-butanol), AEA (remaining aqueous mother liquor) fractions, and MEA (methanol).

**Figure 4:**
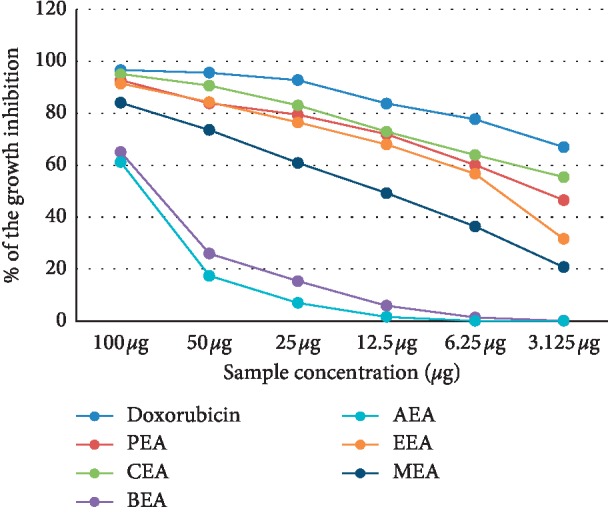
Percentage inhibition of cell growth of *D. dichotoma* extracts against the PC-3 cell line. PEA (petroleum ether), CEA (chloroform), EEA (ethyl acetate), BEA ((n)-butanol), AEA (remaining aqueous mother liquor) fractions, and MEA (methanol).

**Figure 5:**
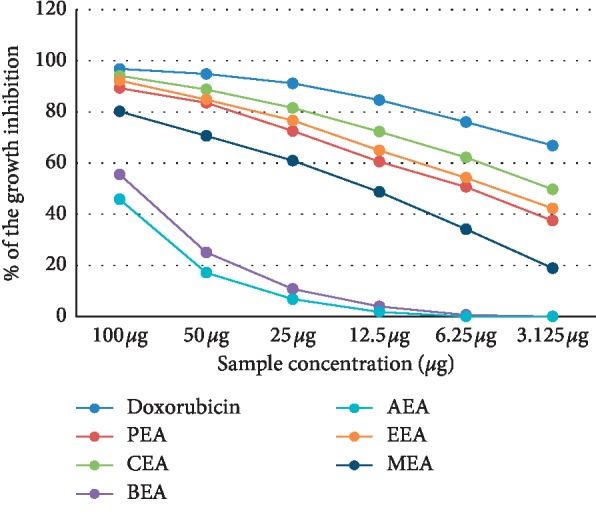
Percentage inhibition of cell growth of *D. dichotoma* extracts against the A-549 cell line. PEA (petroleum ether), CEA (chloroform), EEA (ethyl acetate), BEA ((n)-butanol), AEA (remaining aqueous mother liquor) fractions, and MEA (methanol).

**Figure 6:**
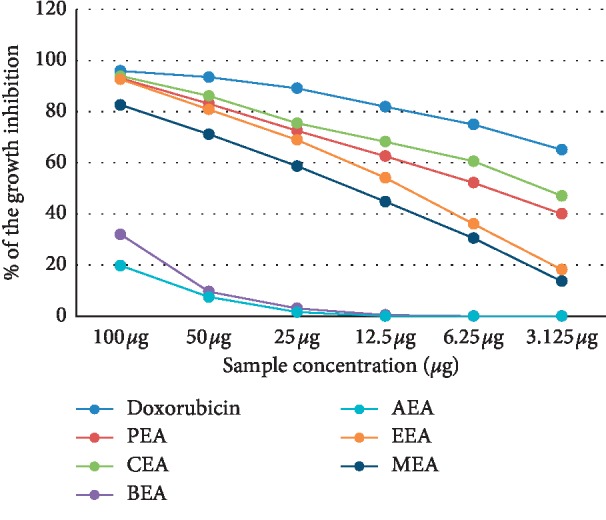
Percentage inhibition of cell growth of *D. dichotoma* extracts against the HeLa cell line. PEA (petroleum ether), CEA (chloroform), EEA (ethyl acetate), BEA ((n)-butanol), AEA (remaining aqueous mother liquor) fractions, and MEA (methanol).

**Figure 7:**
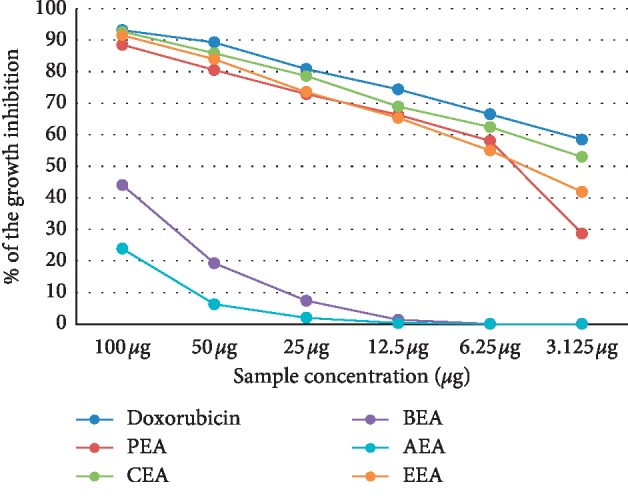
Percentage inhibition of cell growth of *D. dichotoma* extracts against the CACO cell line. PEA (petroleum ether), CEA (chloroform), EEA (ethyl acetate), BEA ((n)-butanol), AEA (remaining aqueous mother liquor) fractions, and MEA (methanol).

The cytotoxicity of different *D. dichotoma* fractions (chloroform, petroleum ether, ethyl acetate, n-butanol and aqueous) against seven cancerous cell lines (HCT-116, MCF-7, HepG2, A-549, PC-3, HeLa, and CACO) was assessed using crystal violet staining viability assay and evaluated in accordance with the guidelines of the American Cancer Institute [24].  Table 1 and Figures 1–7 illustrate the findings and reveal that the less-polar fractions (petroleum ether, chloroform, and ethyl acetate) exerted a significant dose-dependent cytotoxic effect to all seven cell lines while the more-polar fractions (n-butanol and aqueous) did not. The IC_50_ values also indicate that the chloroform fraction displayed the highest overall cytotoxic activity against all seven cancerous cell lines, followed by the petroleum ether and the ethyl acetate fractions. The chloroformic fraction was most effective on MCF-7, PC3, and CACO cell lines (IC_50_ = 1.93 ± 0.25, 2.2 ± 0.18, and 2.71 ± 0.53 *μ*g/mL, respectively) (Table 1). The petroleum ether fraction was also most effective against PC-3 and MCF-7 cell lines (IC_50_ = 3.93 ± 0.51 and 4.77 ± 0.51 *μ*g/mL, respectively). The ethyl acetate fraction, however, showed almost identical effects against six cell lines (IC_50_ of∼5 *μ*g/mL) but was clearly less effective against the HeLa cell line (IC_50_ = 11.1 ± 0.5 *μ*g/mL) (Table 1). It is noteworthy that the effective fractions exerted some degree of selective cytotoxicity indicating that the anticancer effect of the *D. dichotoma* extracts varies depending on the type of cancer being targeted. It is an expected behavior that the same stimuli might produce different reactions when applied to different cancer cells. At the same time, cold extraction may result in a different composition protecting heat-sensitive compounds compared to room temperature extraction [25].

Earlier studies reported that the crude extract of *D. dichotoma* exhibited strong cytotoxicity to various human cancer cell lines including Daudi, Jurkat, HEp-2, and K562 (human leukemic cell) and demonstrated the cytotoxic activity of *D. dichotoma* to be superior to that of other species of brown algae including *Bifurcaria bifurcata*, *Cystoseira tamariscifolia*, *Fucus ceranoides*, and *Halidrys siliquosa* [15, 26]. In fact, *D. dichotoma* has been shown to be a potent cancer inhibitor as it exhibited a remarkably low IC_50_ value of 0.6 *μ*g/mL against MCF-7 cells [16]. Other species of brown algae including *Himantothallus grandifolius* suppressed proliferation and promoted apoptosis-mediated cell death in various epithelial tumor cell lines (i.e., A-549, A-375, HEp-2, and HeLa) [27]. *Sargassum oligocystum* inhibited proliferation of Daudi and K562 cancer cell lines [28], and *Fucus evanescens* not only possessed antitumor effect but also potentiated the antimetastatic activity of cyclophosphamide in mice transplanted with lung adenocarcinoma [29].

In accordance with the selective cytotoxicity displayed by different *D. dichotoma* fractions, an earlier study showed that the chloroform and the ethanol extracts of *D. dichotoma* were more cytotoxic against NCI-H292 cells (human lung mucoepidermoid carcinoma). On the other hand, the methanolic extract exerted higher cytotoxicity to HEp-2 cells while the dichloromethane extract was most active on K562 cells (human chronic myelocytic leukemia) [26]. This selective toxicity was also observed with other marine algae, whereby the dichloromethane extract and chloroform fraction of *Hypnea musciformis* were most cytotoxic to K562 and the chloroform fraction of *P. gymnospora* was more cytotoxic against HEp-2, while the chloroform fraction of *H. musciformis* exerted a more pronounced effect against NCI-H292 [26]. Recently, *D. dichotoma* demonstrated maximum anticancer activity with an IC_50_ of 17.3 ng·mL^−1^ which is very low compared to all other extracts [23]. These data along with our findings suggest that the cytotoxic activity of *D. dichotoma* arises from the activities of various compounds with different cytotoxic properties and a different selectivity towards specific types of cancer. The cytotoxic activity of the extracts could be caused by different mechanisms: either through the activation of an apoptotic route or through a cytostatic effect that stops the cellular cycle. The identification of the mechanisms involved in the cytotoxicity generated by our extracts requires further research.

In our study, the cytotoxic activity was mainly observed in the less-polar fractions of the *D. dichotoma* methanolic extract, with the chloroform fraction exhibiting the highest cytotoxic activity followed by the petroleum ether and the ethyl acetate fractions, with the aqueous fraction being much less effective. Although the compounds responsible for the biological activity of the *D. dichotoma* have not been identified in this study, the presence of several secondary metabolites with low polarities and high ability to penetrate easily through cell membranes is most probably accountable for the anticancer activity [30]. Our results, therefore, are in support of the lipophilic nature of *D. dichotoma* cytotoxic agents, whereby the cytotoxic activity of several algae was attributed to the presence of several nonpolar compounds particularly in the chloroform fraction [26, 30–32]. Other studies also showed that the nonpolar petroleum ether extract [33] as well as dichloromethane [34] of the algae were significantly effective against proliferating cells.

Although the exact cytotoxic constituents and their relative contributions to the anticancer activity of *D. dichotoma* are yet to be elucidated, many researchers believe that the wide array of diterpenes found in brown algae are involved [16, 34–36]. Hydroazulene diterpenes isolated from *D. dichotoma* were reported to be significantly cytotoxic to the cancerous murine cell line KA3IT [33]. Several diterpenes were also isolated from *D. dichotoma* (pachydictyols A–C, dictyol E, cis-africanan-1*α*-ol, and fucosterol) and found to possess cytotoxic activities [16]. Two other cytotoxic diterpenoids in *D. dichotoma*, namely, amijiol acetate and dolastane amijiol-7-10-diacetate, were identified and proved their efficacy against cancer cell lines such as HepG2, and MCF-7 [36].

Along with diterpenes, polysaccharides in brown algae are also believed to play a major role in their anticancer activity through various mechanisms [29, 37], whereby the polysaccharide fucoidan promoted apoptosis in HCT-116 [37] and in AGS (human gastric adenocarcinoma cells) [38]. The polysaccharide ascophyllan isolated from the brown algae *Ascophyllum nodosum* was demonstrated to affect cellular proliferation of U937 cancer cell line in a concentration-dependent manner [39]. Polysaccharide isolated from brown algae, *Heterofucan* SF-1.5V, promotes apoptosis of cancer cells through releasing the apoptosis-inducing factor from the mitochondria into the cytosol [40] and that from *Sargassum latifolium* prevented cancer initiation *via* protective modulation of carcinogen metabolism and cancer antipromoting activity [41], by inducing the carcinogen detoxification enzymes glutathione-S-transferases and decreasing DNA damage as well as enhancing proliferation of macrophages and reducing inflammation involved in cancer promotion.

In addition, other compounds like carotenoids (particularly fucoxanthin) and bromophenols have been involved in the anticancer potential of brown algae [42, 43] being effective in combating skin, duodenal, sarcoma, melanoma, neuroblastoma, hepatoma, leukemia, colon carcinoma, prostate cancer, and urinary bladder cancers in mice [44, 45]. Fucoxanthin, in *D. dichotoma*, has been shown to enhance the expression of proapoptotic caspase-3 and decreased the expression of antiapoptotic bcl-2, survivin, vascular endothelial growth factor, epidermal growth factor receptor, and STAT3 (Signal transducer and activator of transcription 3) [44]. Fucoxanthin also decreased the expression of phosphorylated-Rb (retinoblastoma protein), cyclin D (1 and 2), and cyclin-dependent kinase 4 while it upregulated the expression of p15 (INK4B) and p27 (Kip1) [45]. Carotenoids in brown algae also contribute to the cytotoxic effect via various transcriptional and translational alterations leading to antioxidant, antiproliferative, proapoptotic, and antimetastatic effects [46, 47]. An alternative mechanism by which brown algae are beneficial in cancer treatment is through their ability to enhance the immune system through several mechanisms such as activation of the complement system and promoting phagocytosis of macrophages [48].

### 3.2. Antioxidant Activity

The DPPH radical scavenging activity assay on the different fractions of *D. dichotoma* revealed a weak antioxidant activity as compared to that of ascorbic acid (13.9 ± 0.3 *μ*g/mL) which served as the positive control.  Table 2 illustrates the antioxidant activity of different *D. dichotoma* fractions and shows the crude methanolic extract to have a modest activity (IC_50_ 204.60 ± 8.30) as compared to ascorbic acid. Petroleum ether, chloroform, ethyl acetate, n-butanol, and aqueous extracts exhibited an even weaker antioxidant activity, with petroleum ether and chloroform fractions exhibiting the least antioxidant activity of all the fractions tested. The differences in the extracts' antioxidant capacity depend on the complexity of their composition, which influences their bioactivities [49]. Interestingly, both petroleum ether and chloroform fractions that showed strong cytotoxic activity exhibited the least antioxidant activity. Our results are in line with a recent study showing that the anticancer and antioxidant capabilities of *D. dichotoma* were not correlated well [23]. A more recent study also revealed that high antioxidant activities are not always linked to the highest cytoprotective effect against oxidative stress conditions, suggesting different cytoprotection mechanisms involved in different kinds of antioxidant molecules [50]. Therefore, our results suggest that the prooxidant behavior of antioxidants is not sufficient to explain the anticancer activities of the extracts. While there are a number of studies focusing on growth inhibitory potential of antioxidant compounds [12, 51], there are others claiming a protective role for those substances as cancer cells are known to require antioxidants for their survival [52, 53]. Oxidative stress is one of the most common inducers of carcinogenesis [54]; however, it is also known that once the transformation is completed, cancer cells utilize antioxidant machinery to prevent further damage caused by reactive oxygen species [55]. The protection of the cells by antioxidant components of the extracts may explain the lack of correlation between antioxidant and anticancer capacities of the extracts in our study.

Unlike our study, others have previously reported the antioxidant potential of brown algae including *D. dichotoma* to exhibit superior antioxidant activity to that of red and green algae [56]. The crude extract of *D. dichotoma* was shown to display a significant antioxidant activity as assessed by the PPH radical scavenging activity and by the reducing activity test [15, 46] and attributed the antioxidant activity to their diterpenoids, phenols, phlorotannins, vitamin C, vitamin E, and carotenoids contents [15, 36, 57]. Diterpenoids within *D. dichotoma* were reported to exert a potent antioxidant effect as assessed by the ABTS and erythrocytes hemolysis assays [36], whereas fucoxanthin was an effective radical scavenger, exhibiting 13.5 times higher hydroxyl radical scavenging activity compared to that of vitamin E [46]. With the reported lack of correlation between the various methods used to evaluate the antioxidant capabilities of extracts [34], the use of DPPH assay on its own in our study limits the ability to propose a perspective of the mechanism of the observed modest antioxidant activity of our crude extract.

### 3.3. Acute Toxicity

Our results showed that methanolic extract of *D. dichotoma* was highly well tolerated and safe with even a large oral dose of 5000 mg/kg (data not shown). The animals did not exhibit any noticeable adverse effects or toxicities in the short term. Not a single mortality case occurred in rats within the observation period following the administration of the extract. No changes on the skin or the fur of animals were noticed during the monitoring period. Also, salivation, bowel movement, sleep, and physical activity were all normal even at the high doses administered. In addition, no behavioral changes, consciousness defects, or comas were seen in the tested animals, thus, indicating that the lethal dose 50 (LD_50_) of *D. dichotoma* is significantly high *in vivo* (well above 5000 mg/kg body weight). Along the same line, the acute toxicity of the brown algae *Turbinaria conoides* reported that oral ingestion of a dose up to 5 g/kg of methanol and ethanol-water (1 : 1) extracts was highly nontoxic and did not cause single mortality in rats [58].

## 4. Conclusion

The results presented represent the first significant assessment of the cytotoxicity, antioxidant activity, and safety of Yemeni brown algae *D. dichotoma*. The results indicated that *D. dichotoma* exhibits a potential safe anticancer marine product. Several *D. dichotoma* fractions displayed significant cytotoxic effect on multiple cancer cell lines in a concentration-dependent manner, with the chloroform fraction being the most effective followed by petroleum ether and ethyl acetate fractions. The different fractions of *D. dichotoma*, however, exhibited a weak antioxidant activity, with petroleum ether and chloroform fractions exhibiting the least antioxidant activity. Our results thus imply that the antioxidant capabilities and anticancer activities of *D. dichotoma* do not correlate well and that the prooxidant activity of antioxidants is not sufficient to explain anticancer activities of the extracts. The results also showed that even high doses of the algal extract were highly well tolerated and caused no toxicity signs in rats. Further research will be aimed at pinpointing, characterizing, and isolating the major bioactive substances as well as deciphering their precise mechanisms of cytotoxicity.

## Figures and Tables

**Table 1:** *In vitro* cytotoxic activities of *D. dichotoma* extracts against various carcinoma cell lines.

Cell lines	Doxorubicin	Crude methanolic	Petroleum ether	Chloroform	Ethyl acetate	n-butanol	Aqueous
HCT-116	0.49 ± 0.04	22.80 ± 0.90	5.32 ± 0.24	3.11 ± 0.45	5.50 ± 0.24	86.00 ± 2.40	>100
MCF-7	0.35 ± 0.02	11.00 ± 0.60	4.77 ± 0.51	1.93 ± 0.25	5.33 ± 0.51	70.50 ± 3.40	84.60 ± 1.90
HepG2	0.36 ± 0.02	7.70 ± 0.50	9.31 ± 0.67	2.95 ± 0.43	5.06 ± 0.21	41.20 ± 0.98	79.00 ± 2.80
A-549	0.95 ± 0.16	13.80 ± 0.80	6.09 ± 0.37	3.19 ± 0.58	5.14 ± 0.63	90.90 ± 2.70	>100
PC-3	1.68 ± 0.15	13.40 ± 0.70	3.93 ± 0.51	2.2 ± 0.18	5.52 ± 0.37	80.80 ± 1.40	87.20 ± 2.30
HeLa	3.56 ± 0.12	17.20 ± 0.90	5.68 ± 0.42	3.8 ± 0.14	11.10 ± 0.50	>100	>100
CACO	1.71 ± 0.03	—	5.39 ± 0.24	2.71 ± 0.53	5.06 ± 0.23	>100	>100

Cytotoxic activity is expressed as IC_50_ (*μ*g/mL) ± SD (*n* = 3), which is the concentration of extract at which 50% of cell growth was inhibited relative to cells incubated in the presence of <0.1% DMSO vehicle control. All 7 cell lines were treated with doxorubicin as a positive control. HCT-116 (colon cancer), MCF-7 (breast cancer), HepG2 (hepatocellular carcinoma), A-549 (lung adenocarcinoma), PC-3 (prostate cancer), HeLa (cervical cancer), and CACO (intestinal cancer).

**Table 2:** *In vitro* antioxidant activity of *D. dichotoma* extracts.

Extracts	Antioxidant activity
Ascorbic acid	13.80 ± 0.40
Crude methanolic	204.60 ± 8.30
Petroleum ether	>2000
Chloroform	>2000
Ethyl acetate	211 ± 5.80
n-butanol	1239 ± 28.70
Aqueous	493.20 ± 14.60

Antioxidant activity is expressed as IC_50_ (*μ*g/mL) ± SD (*n* = 3).

## Data Availability

The data set generated and/or analyzed during this study are included in this submitted manuscript and is available from the corresponding author on reasonable request.

## Abbreviations

MEA: Methanol extract of algae
PEA: Petroleum ether extract of algae
CEA: Chloroform extract of algae
EEA: Ethyl acetate extract of algae
BEA: n-butanol extract of algae
AEA: Aqueous extract of algae
HCT-116: Colon cancer
MCF-7: Breast cancer
HepG2: Hepatocellular carcinoma
A-549: Lung adenocarcinoma
PC-3: Prostate cancer
HeLa: Cervical cancer
HEp-2: Human larynx epithelial carcinoma
CACO: Intestinal cancer
DPPH: 2,2-diphenyl-1-picrylhydrazyl
SD: Standard deviation
SPSS: Statistically package for social sciences
IC_50_: The half maximal inhibitory concentration
K562: Human leukemic cell
NCI-H292: Human lung mucoepidermoid carcinoma
STAT3: *Signal* transducer and activator of transcription 3
LD_50_: Lethal dose 50.
